# Evaluation of a modified community based care transitions model to reduce costs and improve outcomes

**DOI:** 10.1186/1471-2318-13-94

**Published:** 2013-09-12

**Authors:** Melanie D Logue, Jennifer Drago

**Affiliations:** 1 University of Arizona, Tucson, Arizona, USA; 2 Sun Health, Sun City, Arizona, USA

**Keywords:** Care transitions, Readmissions, Care coordination, Community-based organizations, Medicare

## Abstract

**Background:**

The Affordable Care Act of 2010 proposed maximum penalty equal to 1% of regular Medicare reimbursements which prompted change in how hospitals regard 30-day readmissions. While several hospital to home transitional care models demonstrated a reduction in readmissions and cost savings, programs adapted to population needs and existing resources was essential.

**Methods:**

Focusing on process and outcomes evaluation, a retrospective analysis of a modified community based care transitions program was conducted.

**Results:**

In addition to high levels of patient satisfaction with the care transitions program, participants’ confidence with self care was significantly improved. Further, the program evaluation demonstrated a 73% reduction in readmissions and an actual Medicare cost savings during the 9-month study period of $214,192, excluding the cost to administer the program.

**Conclusions:**

While there are several transitional care programs in existence, a customized approach is desirable and often required as the most cost effective way to manage care transitions and employ evidence based policy making. This study established some of the pitfalls when implementing a community-based transitional care program and demonstrated encouraging outcomes.

## Background

Acute care hospitalizations have consistently accounted for the largest portion of total Medicare expenditures hovering around 27% over the last few years [1-4]. In a recent study, Jencks et al. [5] found that one in five acute care hospitalizations in the United States result in readmission within 30 days which stimulated national discussion on poor quality care as a root cause. The Centers for Medicare and Medicare Services (CMS) began publicly reporting hospitals’ 30-day readmission rates for Medicare beneficiaries discharged for congestive heart failure, acute myocardial infarction, and pneumonia [6]. The Medicare Payment Advisory Commission (MedPAC) estimated that the costs associated with 30-day hospital readmissions accounted for approximately $15 billion in annual Medicare spending, with the majority of those costs attributable to preventable causes [7]. Medicare’s Hospital Readmission Reduction Program, resulting from the 2010 Affordable Care Act, proposed penalties for those hospitals with excessively high readmission rates for heart attack, heart failure, and pneumonia [8]. With a proposed maximum penalty equal to 1% of regular Medicare reimbursements, this law stimulated change in how hospitals regard 30-day readmissions and they examined factors that contributed to them. Hospitals began to identify phenomena that contributed to a post-discharge period of vulnerability. Shorter hospital stays and an increased acuity of illness paired with higher expectations for self-care, low health literacy, and cognitive impairments adds to the vulnerability associated with being discharged from an acute care facility [9]; while receiving quality care during the initial hospitalization, effective discharge planning, coordinated post-hospital care, and timely follow-up can prevent readmission [10]. The problem of 30-day readmissions is worldwide, however. In one study comparing international readmission for acute myocardial infarctions, the 30-day readmission rate was 7.7% in non-U.S. countries including Canada, Australia, New Zealand, and 13 European countries [11]. Another study examining international hospital readmissions found that shorter stays correlated with higher readmission rates and vice versa [12]. Explanations can be sought in differences among healthcare systems and policies.

Needing to improve the current state of post-discharge care, researchers explored effective delivery models to assist in these transitions of care from the hospital to home addressing the gaps in care that occur. Several studies in transitional care have shown that properly designed and executed programs improve quality outcomes and achieve cost savings [13-15]. The Community-Based Care Transitions Program (CCTP), created by the Affordable Care Act, began supporting research designed to test transitional care models to reduce readmissions for beneficiaries and document savings to the Medicare program [16]. Acting as a community liaison, community-based organizations (CBOs) can reduce fragmentation, improve collaboration and coordinate care across settings, and offer additional resources to an expanding aging population with multiple chronic conditions [17]. A community-based organization is a “public or private nonprofit (including a church or religious entity) that is representative of a community or a significant segment of a community, and is engaged in meeting human, educational, environmental, or public safety community needs” [18]. Care coordination involves the sending healthcare team, the receiving healthcare team, and the administrative leadership of the healthcare institution [19]. The USDHHS [17] identified several elements that promote safe and effective care transitions including:

● Patient and/or caregiver training to increase activation and self-care skills.

● Patient-centered care plans that are shared across settings of care.

● Standardized, accurate, and timely communication and information exchange between the transferring and receiving healthcare teams.

● Medication reconciliation and safe medication practices.

● Ensured transportation for health care-related travel.

● Procurement and timely delivery of durable medical equipment.

● Ensuring the sending healthcare team maintains responsibility for care of the patient until the receiving healthcare team confirms the transfer and assumes responsibility ^(¶12)^.

Identified proxies for inadequate care transitions include medication errors, increased health care utilization, redundancies in care, inadequate patient and/or staff preparation, family and/or caregiver stress, inadequate follow-up care, litigation, and dissatisfaction [19]. Aspects of care that are critical to a successful care transition include information transfer, a plan to target at-risk patients, reimbursement incentives aligned with transitional care, new models of care delivery, and best practices for transitional care [18]. Here, we describe an Arizona CBO’s care transition program, present program evaluation findings, and discuss the implications and lessons learned from this experience.

### System description

Sun Health, an Arizona CBO, has longstanding relationships with nearby healthcare institutions and provides community support programs including senior living services, philanthropic support, medication management, community education, and other programs to support healthy senior living. In November 2011, Sun Health launched a care transitions program in collaboration with two area hospitals. The two hospitals belong to a larger nonprofit health system that Sun Health is neither operationally nor financially related to, but whom approached Sun Health as a community partner. Before developing and implementing a care transitions program, the scope of the problem was defined using the STate Action on Avoidable Rehospitalizations Readmission (STAAR) Diagnostic Review tool [20] as part of a system-wide assessment. The qualitative and quantitative root cause analysis included chart reviews and interviews with patients, caregivers, and providers across care settings who were involved in the circumstances leading up to readmission. Key findings of the diagnostic review are presented here. Additional research performed at both hospitals revealed no clear pattern of readmissions based on discharge or admission day of the week, attending physician or PCP.

In 2010, Hospital A had 6,478 Medicare FFS readmissions among 4,732 individual patients. The all-cause unadjusted 30-day readmission rate was 14.7% (908/6148). Fifty percent (470) of the 30-day readmissions occurred within 10 days of discharge; 15% of those (131) occurred within 48 hours; 20% (178) occurred within 72 hours; and 25% (235) occurred within 4 days of discharge. Fourteen diagnoses accounted for 25% (242/908) of readmissions. The top primary diagnoses by volume included heart failure, renal failure, urinary tract infection, gastrointestinal bleeding, anemia, sepsis, arrhythmia, chest pain, gastritis, esophagitis, syncope, pneumonia, respiratory infection, and chronic obstructive pulmonary disease.

In the same year, Hospital B had 8,586 Medicare FFS admissions among 6,064 individual patients. The all-cause unadjusted 30-day readmission rate was 15.4% (1276/8298). Fifty percent (588) of the 30-day readmissions occurred within 10 days of discharge and 25% (294) occurred within 4 days of discharge. The same fourteen diagnoses accounted for a similar percentage of readmissions with the addition of stent placement (29/188) relative to the expertise of Hospital B. Additional evaluation of both hospitals revealed no clear pattern of readmissions based on discharge or admission day of the week, attending physician, or primary care provider.

Chart reviews were completed on ten patients from Hospital A and B, for a total of 20. Family and provider interviews were conducted on the same 20 readmissions. The following four themes were observed from those interviews: (1) lack of availability of physician follow up visits within seven (7) days of discharge; (2) late discharges contributing to patient fatigue, hindering comprehension of discharge instructions and ability to get prescriptions filled; (3) medication issues (prescriptions not filled, incomplete patient instructions, duplicate therapies prescribed, non-adherence); and (4) vague discharge instructions and/or patient education materials. Patients reported feeling overwhelmed with new information, were uncertain about next steps in the plan of care, and needed additional support in the post-discharge period.

Based upon program results from the CTI [13] and consistent with the Partnership for Patients [21], the initial goal of the Sun Health care transitions program was to reduce the expected readmission rate of 19.7% among a population of 1,800 patients by 20%, which would prevent 72 readmissions annually. Using the Medicare FFS data provided on the Institute of Medicine Hospital Referral Region (HRR) [22] the average cost of a Medicare hospitalization in the identified service area is $13,387; thus, this program aims to avoid $963,789 in annual costs attributed to avoided hospitalizations for those 72 patients. The blended rate (including in-hospital and program nurses’ time, mileage, supervision, and materials) for the Sun Health care transitions program administration services was estimated at $360 per discharge. The total cost of the Sun Health care transitions program is $648,000 for 1,800 patients; thus, this program potentially offers CMS a net savings of −30% or $288,864 (difference between annual cost savings and cost to administer the program).

Along with this diagnostic review, the literature on care transition interventions was reviewed and used to develop a customized program to adequately service the study population using existing institutional-based interventions for at-risk Medicare fee-for-service (FFS) beneficiaries discharged to home without skilled home care services. The Sun Health care transitions program was modeled after the Coleman Transition Intervention (CTI) [13], Naylor’s Transitional Care Model (TCM) [14,15], and other national recommendations describing essential components of safe and effective care transitions [17,19]. The Sun Health care transitions program targeted the three diagnoses indicated by the Hospitals Readmissions Reductions Program [16] and then expanded to other diagnoses based on the diagnostic review conducted by the CBO. Because an all-RN model was cost-prohibitive, the Sun Health care transitions program employed licensed practical nurses (LPNs) to enroll patients and coordinate care, and employed registered nurses (RNs) to make home visits. Program nurses worked with both hospitals’ nurse case managers (CMs) to identify potential program enrollees. The aims of the care transitions program were to (1) educate patients about their health condition, including red flags, and teach self-monitoring of chronic disease; (2) perform a medication reconciliation and create an up to date medication list; (3) ensure timely physician follow up; (4) provide a patient-centered health record including a plan of care for their recovery; and (5) assess the need for other supportive community resources such as home-delivered meals, volunteer transportation to medical appointments or medication assistance.

### Program description

**Figure 1:**
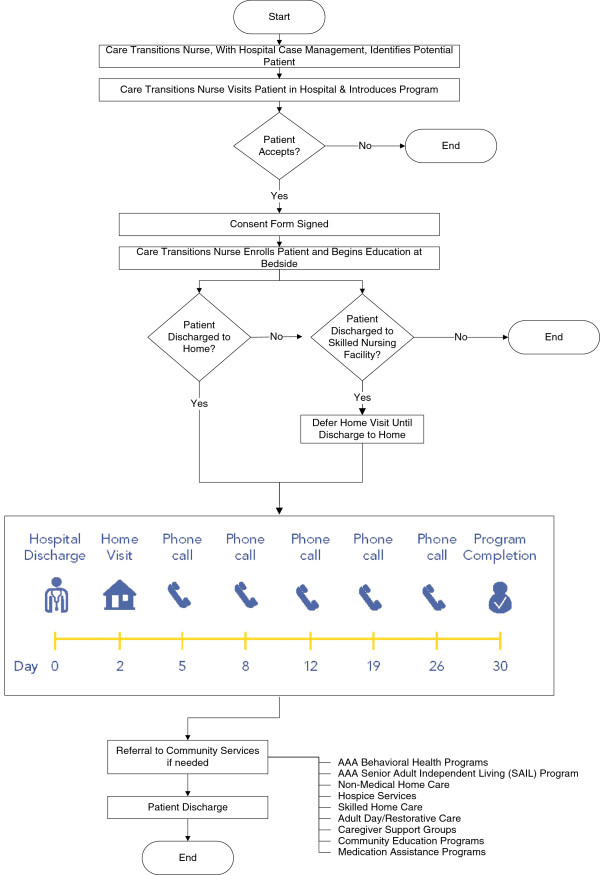
Sun health care transitions program model.

Sun Health transitional care program nurses were trained by a local Center for Aging on best practices in care transitions and geriatric specific screening tools. Sun Health coordinated the care transitions program and extended case management services into the community where the Sun Health transitional care nurses follows them home and ensures continuity of care. The program began with 3 RNs and 1 LPN. The nurse to patient staffing ratio was high in the beginning, so the intervention was very manageable. As the program expands, RN/LPN teams will manage a case load of 60 patients per month. Identification of planned discharges was initiated internally by the two hospitals who queried their electronic record systems daily. Utilizing this query as a starting point, Sun Health’s LPN, acting as in-hospital coaches, rounded daily at both hospitals and interfaced with designated CMs to review potential cases for inclusion in the care transitions program. Written materials developed by the CBO were available to share with patients as they considered participating in the care transitions program. Similar to the CTI, the Sun Health care transitions program focused on four key areas including: (1) medication self-management, (2) the use of a paper-based personal health record (PHR) by the patient or caregiver to facilitate communication and ensure continuity of the care plan across providers and settings, (3) ensuring timely follow-up visits with the receiving care teams, and (4) educating patients on red flags indicating worsening of their condition. The Sun Health care transitions program was also expanded to include depression and mobility screening using the PHQ-2 [23] and the timed ‘Up and Go’ test, respectively [24]. Patients received an initial phone call on the day of discharge or the day following discharge to set up their initial home visit within 24 to 48 hours. A home visit by the RN occurred, ideally, within 24–48 hours post-discharge. The initial home visit was then followed by three to five phone calls at various times during the 30-day post-discharge period, depending on the needs of the patient (Figure 1). During the 30-day program, patients learned self-management skills and received customized tools to actively engage them in their care.

The initial home visit was where the care transitions program nurses addressed patient and/or caregiver training, developed patient-centered care plans, reconciled medications and provided medication education, ensured timely follow-up appointment were in place and arranged transportation as needed, and ensured that the patient and/or caregiver had necessary resources in place. Program teaching materials were identical to those used by the discharging institutions and helped to reinforce the patients’ learning. “Stoplight” diagrams illustrated condition red flags and when to contact a physician. Family and caregiver instruction were also provided. An assessment of the patient’s need for community based resources during the initial home visit and subsequent telephone interactions was performed. Referrals were made to available free or low-cost resources in the catchment area including home-delivered meals, medication assistance programs, medical equipment loan closets, volunteer transportation to medical appointments, volunteer grocery shopping, volunteer home maintenance, friendly visitor, telephone reassurance, and financial assistance programs. Referrals to post-hospital care services such as personal care/attendant services, geriatric case management services, skilled home care and hospice care were also available if needed. Patients were discharged from the program after they completed the 30 day post-hospitalization program or if they elected to end services sooner. Discharge summaries were written by the RNs and kept in the patient’s encounter form and were shared with the patient’s PCP.

## Methods

### Design

Focusing on process and outcomes evaluation, the following research questions were addressed:

1. Was the piloted care transitions program administered as planned?

2. Did the program improve acute care readmission rates?

3. Were patients satisfied with the program?

For this retrospective analysis, data from persons participating in the Sun Health care transitions program was analyzed. The process evaluation examining whether the care transitions program was administered as planned was conducted after the first 24 patients completed the program. This cut-off point was selected to coincide with a transition in patient encounter documentation from Word files to Web-based files and the expansion of the program to include diagnoses beyond the original three targeted by CMS. The outcome evaluation was conducted at the end of July 2012 (9 months after the program was initiated) and include 149 patients.

### Sample and setting

The two previously described facilities have a hospital catchment area that incorporates 14 zip codes and is comprised of 49,694 Medicare FFS beneficiaries. The population in the catchment, a retirement community in Arizona, is rather homogenous. 58.3% are female and 41.7% are male [25]. Of those, 94.4% are Caucasian, 2.8% Hispanic, and 1.4% Black [25]. The mean resident age is 73.4 years and the average household income is $33,632 [25]. Over 86% of the Medicare beneficiaries residing in this area use the two hospitals [26]. The target population for the Sun Health care transitions program is adults who are Medicare FFS beneficiaries with a discharging diagnosis in the top 8 categories (cardiac, respiratory, gastrointestinal, infectious, renal, neurologic, orthopedic, and/or diabetes) identified in the pre-program diagnostic assessment who were discharged from the hospital to home without skilled home care. All eligible patients being discharged from the two participating hospitals (i.e. those with the top primary diagnoses by volume according to the diagnostic review) were selected and enrolled if they consented. However, recruitment for the first 24 patients was limited to the three CMS targets, pneumonia, acute myocardial infarction, and congestive heart failure. Once success was seen with the first 24 patients, the care transitions program was broadened to include other diagnoses to meet the specific needs of the hospitals.

### Instrumentation

Data on program outcomes were collected in two formats: (1) patient encounter forms completed by program nurses and (2) post-program patient satisfaction surveys sent out by the CBO (Additional file 1). Patient encounter forms included data such as the numbers of encounters and the dates on which they occurred. Informed consent for participation in the study was obtained from participants. Caregiver information and level of involvement were also identified. Information on the primary care provider and specialists follow-up appointments and transportation needs were identified, with the goal of the first follow-up being within 7 days of the discharge. Documentation on post-discharge medication reconciliation, delivered medication education, red flags for respective conditions, delivered health education specific to the condition, an action plan for self-management and care, and use of a paper-based personal health record were also recorded in the patient encounter forms. In addition, the encounter forms captured the current number of medications, over the counter agents, and medication adherence along with any history of re-hospitalizations, emergency department visits, or unscheduled follow-ups with clinicians. The results of the depression and mobility screens were also documented in the patient record. Any additional notes on patient progress such as care coordination issues, care conferences, and progress were noted.

Patient satisfaction surveys were developed and administered by Sun Health and mailed to participants upon program completion. The survey began with a letter from a Sun Health representative stating its purpose and contained 22-items using 5-point Likert scales (excellent to poor) asking about their satisfaction with the program nurses, type of communication, education materials, and overall program rating. Pre and post ratings of the program participants’ knowledge and skills related to their current condition were also collected using a 4-point Likert scale with the anchors, very confident to not at all confident. An open-ended question asking patients what they like best and least about the program and a single select multiple choice question about whether they would recommend the program to others completed the survey. Satisfaction surveys did not have any identifiable patient information and were not linkable to program data at any point.

### Procedures

Prior to completing the program evaluation, approval for the study was obtained from the University of Arizona Human Subjects Review Committee. The researcher familiarized herself with the data set and goals of the evaluation including how the data was collected and measured. The targeted program outcome variables identified by the CBO were 30-day readmissions, number of medication discrepancies identified, number of assisted appointments, and averted care visits. Data were collected by the Sun Health care transitions nurses and CMs in the patient encounter forms, and then manually aggregated into an Excel spreadsheet by one nurse according to the outcome variables. The CBO then mailed the patient satisfaction surveys to all program participants who completed the program. Administrative assistants manually aggregated the patient satisfaction surveys results and entered that data into another Excel spreadsheet. The researcher analyzed both sets of data after having confirmed the accuracy of the manual entries by duplicating this process.

### Data analysis

The raw data from the original paper-based records and the case management software program were imported by the researcher into SPSS (SPSS 20, 2011) [27]. SPSS was used to calculate central tendencies and descriptive statistics about program participants and reported in aggregate form. Individual demographic information was not collected or reported since the focus of this study was outcomes. Cross tabulations and Spearman correlations were used to compare readmissions with number of medication discrepancies. Paired samples T-tests were used to compare the pre and post program responses on the patient satisfaction survey regarding participants’ confidence in their knowledge and skills surrounding their condition. Missing data were removed from the calculations and the valid percent was used when reporting results.

## Results

### Process evaluation

Of the 24 patients, 4 patients had the diagnosis of acute MI (16.7%), 19 patients had the diagnosis of CHF (79.2%), and 1 patient had pneumonia (4.2%). Table 1 summarizes patient encounters, medications, and reported caregiver assistance. Most patients (19) had between 1 to 6 follow up calls while in the program (79.2%). Most patients (19) received between 3–8 encounters (79.2%). The variability in number of encounters and medication was attributed to acuity and/or severity of the patient’s condition. The number of visits was at the discretion of the transitional care nurses as long as the minimum program requirements were met. Exclusions occurred where patients requested that the program be discontinued if the patient reported comfort with self-care. Table 2 summarizes the frequency of measured items in the patient encounter forms.

**Table 1:** Descriptive statistics for total number of patient encounters, medications, and reported caregiver assistance

	**Total number of follow-up calls**	**Total number of calls attempted**	**Total number of encounters**	**Total number of home visits**	**Total number of patient medications**	**Reported percent of time caregivers assisted patients**
N Valid	24	24	24	24	12	12
N Missing	0	0	0	0	12	12
Mean	3.79	1.33	6.08	1.75	8.86	71.58
Median	4.00	1.00	5.50	1.50	8.00	92.50
Range	11.00	8.00	14.00	4.00	17.00	90.00

**Table 2:** Frequency of measured items using the original group of patients (N=24)

	**Total Number**	**Percent**
Reported averted care visits (hospital or office)	11	45.8
Appointment assistance	10	41.7
Follow up appointment(s) scheduled	21	87.5
Transportation to appointment(s) in place	20	83.3
Follow up scheduled within 7 days	10	41.7
Follow up scheduled with specialist	19	79.2
Medication reconciliation	21	87.5
Medication education	21	87.5
Education on condition red flags	22	91.7
Education on condition specific information	22	91.7
Received individualized care plan	22	91.7
Hospital A referrals	12	50
Hospital B referrals	12	50
Reported unplanned care visits	5	20.8
Depression screening completed	10	41.7
Mobility screening completed	10	41.7

Two-thirds of all patients (16) had 7 or more medications (66.7%). In a typical week most patients (9) missed, skipped, or had some other medication adherence problem (56.3%). More than half of all patients (14) also took over-the-counter medications (56.3%). Two patients were re-hospitalized within 30 days during the initial program (8.3%), while 22 were not (91.7%). Re-hospitalizations were reported to be averted as a result of nursing interventions in 5 patients (20.8%). Emergency room visits were reported to be averted as a result of nursing intervention in 3 patients (12.5%). Assistance from program nurses was needed in 45.8% of the cases to ensure that the patient had an appointment, had transportation to the appointment, and/or intended to go to the appointment.

Most care participants were the patient themselves (19 or 79.2%), significant others (13 or 54.2%), family members (4 or 16.7%), or other caregivers (6 or 25%). On a scale of 1 (lowest) to 4 (highest), 8 patients rated their participation level a 4 (33.3%), 4 rated their participation level a 3 (16.6%), and 4 rated their participation level a 2 (16.7%). On a scale of 1 (lowest) to 4 (highest), 6 caregivers rated their participation level a 4 (25%), 3 rated their participation level a 3 (12.5%), and 2 rated their participation level a 2 (8.3%). Nine caregivers were reported as living in the home (39.1). The percentage of time caregivers assisted in care ranged between 10-100%. Most (4) were identified as giving 100% of their time. Eleven caregivers had daily contact with the patients (78.6).

### Outcome evaluation

A total of 149 patients, including the original 24, were used in the outcome evaluation. 79 patients were male and 70 were female ranging between the ages of 51–95. Program nurses assisted 52 patients (35.9%) with follow up appointments. A total of 300 medication discrepancies were found by the nurses. The average number of medication discrepancies was 2.1 per patient. Medication discrepancies (Table 3) were not reported for 3 patients. There were also no statistically significant correlations between gender and medication discrepancies (p=.08) or age and medication discrepancies (p=.86).

**Table 3:** Frequency of medication discrepancies

**Number of medication discrepancies**	**Frequency**	**Valid percent**
0	46	31.5
1	36	24.7
2	16	11.0
3	15	10.3
4	10	6.8
5	8	5.5
6	7	4.8
7	3	2.1
8	2	1.4
9	2	1.4
10	1	.7
Total	146	100

The 30-day readmission rate was 4% (6) for patients who completed the program. Patients who were re-admitted as observation status were not included in the readmission final readmission count. Readmission was not correlated with the number of medication discrepancies. Of the 6 readmitted patients, 1 had no medication discrepancies. The other readmitted patients had between 1 and 4 medication discrepancies, but more medication discrepancies did not predict readmission (p=.60). There were also no statistically significant correlations between gender and readmission (p=.13) or age and readmissions (p=.39).

A total of 62 patients completed the patient satisfaction survey. The majority of respondents (58 or 98.3%) rated the nurses’ knowledge very good or excellent. Table 4 summarizes the survey responses. Patients were also asked to recall and rate their pre and post program knowledge, skills, ability to recognize changes in their condition(s), and ability to act on those changes.

**Table 4:** Patient Satisfaction Survey Responses

	**Minimum**	**Maximum**	**Mean**	**SD**
Nurses knowledge	2	5	4.73	.55
Nurses courteousness	4	5	4.83	.38
Nurses care and concern	3	5	4.82	.43
Calls returned promptly	4	5	4.77	.43
Questions were answered	3	5	4.78	.46
Conditions were explained	3	5	4.77	.50
Medications were explained	3	5	4.69	.60
Condition red flags were explained	2	5	4.78	.52
Information was understandable	4	5	4.82	.40
Discussed ways to stay healthy	3	5	4.75	.48
Educational materials	3	5	4.63	.61
Paper-based personal health record	3	5	4.62	.62
Overall rating	4	5	4.82	.39
Confidence in patient’s pre-program knowledge	1	5	3.03	.97
Confidence in patient’s pre-program skills	1	4	2.98	.89
Confidence in ability to recognize change in health status pre-program	1	4	3.07	.94
Confidence in own ability to take appropriate action pre-program	1	4	3.02	.93
Confidence in patient’s post-program skills	2	4	3.58	.62
Confidence in patient’s post-program skills	2	4	3.56	.60
Confidence in ability to recognize change in health status post-program	2	4	3.61	.56
Confidence in own ability to take appropriate action post-program	2	4	3.63	.52

Using paired samples T-tests, differences between the participants’ pre and post confidence ratings of themselves were statistically significant (p < .01) (Table 5) when asked about their confidence in knowledge about their condition, their skills, their ability to recognize changes in their condition, and their ability to act on those changes.

**Table 5:** Pre and post confidence changes using paired samples t-test

**Pairs**	**Mean**	**Confidence interval**	**Significance**
Confidence in program knowledge	-.51	-.73 to-.28	.001
Confidence in program skills	-.54	-.74 to -.35	.001
Confidence in ability to recognize change	-.52	-.73 to -.31	.001
Confidence in ability to take appropriate action	-.62	-.84 to -.39	.001

## Discussion

### Process evaluation

The administration of the care transitions program proceeded accordingly with few departures in the overall process. Occasional outliers were observed in the number of patient care visits or telephone contacts, but the overall numbers of visits remained consistent with the program model (Figure 1). As expected with any new process, some omissions in documentation were observed with large variations from nurse to nurse. These omissions were seen in the areas of documentation of medications and depression and mobility screenings. Some of these omissions were attributed to the original Word formatted documentation tool which was reported as tedious by the care transitions program nurses. Based on that feedback, the documentation method changed to a Web-based case management software program which allowed expanded access to the patient care record across multiple care settings over time. This software eliminated specific areas for charting, such as dropdowns, prompts, and checklists, and contained mostly text boxes. With the changeover to the Web-based software documentation system and the different reporting format it seemed to exacerbate the problem of missing information, however. It also created challenges for the care transitions program nurse to extract data from the software program because it required her to carefully read through each chart and look for specific measures of program outcomes within the text. Because the software program is customizable, dropdowns, prompts, and checklists will be added back in the near future to capture key variables needed for program monitoring along with additional training and ongoing support for program nurses. Additionally, periodic re-education for the program nurses was required based on finding of program adherence and anytime programmatic changes were made.

An observation with visible external influences was the high percentages of scheduled 7 day follow up appointments with the receiving team. The program goal was for 100% of patients to have a timely follow up appt (w/n 7 days). In approximately 50% of the cases, the hospitals made the follow up appt for the patient prior to discharge. Assistance by program nurses was given to another 45.8% of the cases, totaling 95.8%. In a study by Jencks [28], outpatient physician claims were not submitted on behalf of 50% of Medicare patients who were readmitted within 30 days after initial discharge. This finding suggests that post-discharge follow up appointments are completed in half of all discharged patients on Medicare and is consistent with the delays in timely follow up appointments seen in this study. Another study by Sommers and Cunningham [29] found that one-third of discharged patients did not see a healthcare provider in the 30 days following hospital discharge, excluding those seen in emergency departments which points to a substantial gap in care coordination once the patient is discharged. Further, the impact of nursing assessments was evidenced by the numbers of reported averted care visits. For the purposes of this research, averted care was defined as health care visits that were prevented due to nursing assessments and interventions. Nurses made a judgment call as to whether the patient would have sought care without the involvement of the nurse. Definitions or parameters of averted care visits was not defined a priori, however, aided visits included any effort on behalf of the nurse to arrange or ensure a care visit was made to the receiving team. Patients often had their own appointments and transportation to those appointments in place and were commonly identified as active participant in their own care. Levels of involvement in care participation varied, but most program participants identified another person to assist them in their care. A study by Arbage et al [30]. suggests that patients who have caregiver support are at less risk for a hospital readmission than patients who live alone. Finally, polypharmacy and related issues were common in this group. Due to the risk factors of age and comorbidities, this was not surprising and is consistent with the transitional care literature [31,32]. Some additional readmission factors that we noted through patient statements were an inability to connect with an MD in the office (evenings and weekends were problematic), anxiety, lack of attention to red flags, in some cases a lack of compliance with recommendations, and medication adherence. In this study, we did not track the reasons for readmission; however, we suspect multiple factors are involved.

### Outcome evaluation

In addition to high levels of patient satisfaction with the Sun Health care transitions program, participants’ confidence with self care was significantly improved. Further, based on a 15% baseline readmission rate for both hospitals, we would have expected 22 readmissions out of 149 patients enrolled in the Sun Health care transitions program. Our results far exceeded this goal with only 6 patients (4%) who participated in this program being readmitted within the 30-day study period. Since all patients receiving home care were excluded from the sample there may have been some subject bias towards more stable patients, even so, the findings were positive. When compared to the baseline all-cause readmission rate of both hospitals, there was a decrease in 30-day readmissions of 73% (difference between expected and actual readmissions) which surpassed the 20% Medicare goal and the goals of this study. This equals an actual Medicare cost savings during the 9-month study period of $214,192 (comparing baseline rates to study rates), excluding the costs to administer the program. Because information on patient eligibility for the Sun Health care transitions program was unavailable for the baseline readmission rate for both hospitals, program administration costs could not be compared. When matched against the national average for 30-day readmissions (30 expected readmissions out of 149 patients), the Sun Health care transitions program has shown an 80% reduction in readmissions. Medicare costs for 30 readmitted patients would have totaled $401,610; however, with only 6 readmissions at an estimated cost of $80,322, this program demonstrated a savings of $321,288. Specific details on potentially preventable readmission (PPR) [10] calculations were not within the scope of this preliminary evaluation, thus, were omitted in this discussion. However, it is important to consider circumstances that led to readmissions and, for accuracy purposes, calculate PPRs using existing measurement models [33] in the future.

Another notable finding in this evaluation was that while medication discrepancies were prevalent in this population, there was no correlation with readmission. The significance of these findings are that while the care transitions program has shown a reduction in 30 day readmissions and that medication discrepancies play a role, there was no direct relationship to readmissions. A study by Coleman et al. [34] contradicts this finding. In the Coleman study, 14.3% of the patients who experienced medication discrepancies were rehospitalized within 30 days and was reported as statically significant. The incongruity in findings might be attributable to the low sample size in the present study or the lack of a specific tool to measure medication discrepancies, such as the Medication Discrepancy Tool [35].

The clinical application for the current findings are the need to provide ongoing critical examination of program processes and clearly define the parameters and feasibility of data collection for the purposes of program monitoring beforehand. While program planning and research have distinctly different foci, it is worthwhile to consider consultants on the project so that data accuracy and consistency is ensured. Employing a standardized Medication Discrepancy Tool [35] will also ensure consistency among the data. Another clinical consideration is the need for patient data to be documented in an electronic database format so that data pulls become easier to conduct and less prone to human error when measuring outcomes. Paying attention to and developing approaches to reduce bias is also important to avoid skewed results. Finally, ample staff training and/or retraining is often necessary as transitional care programs evolve and lessons are learned in the process.

## Conclusion

While there are several transitional care programs in existence, a customized approach is desirable and often required as the most cost effective way to manage care transitions and employ evidence based policy making. Population characteristics, reasons for readmission, available services, and existing infrastructures and resources should be considered in the program planning phase. As healthcare delivery and reimbursement systems change and gaps in care shift, so must the approach to care. Using evaluation methods to assure program efficiency, effectiveness, quality and value are crucial in an accountable care environment. As accountable care organizations enter the marketplace and responsibility for patient outcomes are shared among the sending and receiving teams, CBOs are well positioned to act as a liaison between the inpatient and outpatient care settings by drawing upon their strengths and capacities as community-oriented institutions. Positioned as experts in home-based care and resources, partnerships with CBOs stand to fill the gap from hospital discharge to ambulatory follow-up care. This study established some of the pitfalls when implementing a community-based transitional care program and demonstrated encouraging outcomes. Ongoing comparative effectiveness and outcome research establishing ideal transitional care models is prudent as we move forward in our understanding and enhancement of the 30-day hospital readmission phenomena. Because there is no control group in evaluation research, causal links could not be established between variables. Although this study demonstrates efficacy, further translational studies would need to be conducted to determine program effectiveness in other settings. Future research should also include tracking and reporting patient risk profile to guide program replication and allocation of resources.

## Competing interests

The authors declare that they have no competing interests.

## Authors’ contributions

Both authors have made substantial contributions to conception and design, or acquisition of data, or analysis and interpretation of data; have been involved in drafting the manuscript or revising it critically for important intellectual content; and have given final approval of the version to be published. Both authors read and approved the final manuscript.

## Authors’ information

ML is a family nurse practitioner in a private practice and a clinical assistant professor at the University of Arizona. She is currently completing a post-doc in comparative outcomes and clinical effectiveness there. ML has served as a consultant to Sun Health working on the transitional care program with JD. JD is vice president of business development at Sun Health and is responsible for developing and launching community-based health programs, such as the Sun Health Transitional Care Program.

## Pre-publication history

The pre-publication history for this paper can be accessed here:

http://www.biomedcentral.com/1471-2318/13/94/prepub

## Supplementary Material

Additional file 1 Patient satisfaction survey. Click here for file
