# Supplementary material for: Evaluation of a modified community based care transitions model to reduce costs and improve outcomes
Source: BMC Geriatr. 2013 Sep 12;13:94. doi: 10.1186/1471-2318-13-94 (PMC3848703; doi:10.1186/1471-2318-13-94)
Supplement: Additional file 1 — Patient satisfaction survey. [file 1471-2318-13-94-S1.docx]

**PLEASE RATE THE FOLLOWING: Your responses will be kept strictly confidential.**

|  | **Excellent** | | **Very Good** | | **Good** | | | **Fair** | | | **Poor** | | **Does Not**  **Apply** | |
| --- | --- | --- | --- | --- | --- | --- | --- | --- | --- | --- | --- | --- | --- | --- |
| **Our Nurses** |  | |  | |  | | |  | | |  | |  | |
| Nurse’s Knowledge Level/Expertise | 5 | | 4 | | 3 | | | 2 | | | 1 | | N/A | |
| Nurse’s Courteousness | 5 | | 4 | | 3 | | | 2 | | | 1 | | N/A | |
| Nurse’s Caring and Concern | 5 | | 4 | | 3 | | | 2 | | | 1 | | N/A | |
|  |  | |  | |  | | |  | | |  | |  | |
| **Our Communication With You** |  | |  | |  | | |  | | |  | |  | |
| How promptly we returned calls | 5 | | 4 | | 3 | | | 2 | | | 1 | | N/A | |
| How well we answered your questions | 5 | | 4 | | 3 | | | 2 | | | 1 | | N/A | |
| Our explanation of your condition | 5 | | 4 | | 3 | | | 2 | | | 1 | | N/A | |
| Our explanation of your medications and assistance with creating medication list | 5 | | 4 | | 3 | | | 2 | | | 1 | | N/A | |
| Our explanation of red flags/warning signs | 5 | | 4 | | 3 | | | 2 | | | 1 | | N/A | |
| Our ability to explain things in a way you could understand | 5 | | 4 | | 3 | | | 2 | | | 1 | | N/A | |
| Advice given to you on ways to stay healthy | 5 | | 4 | | 3 | | | 2 | | | 1 | | N/A | |
|  |  | |  | |  | | |  | | |  | |  | |
| **Educational Materials Provided** |  | |  | |  | | |  | | |  | |  | |
| Effectiveness of educational materials provided to you | 5 | | 4 | | 3 | | | 2 | | | 1 | | N/A | |
| Usefulness of Patient Care Record binder | 5 | | 4 | | 3 | | | 2 | | | 1 | | N/A | |
|  | | | | | | | | | | | | | | |
| **Your Overall Rating of the Sun Health Care Transitions Program** | | 5 | 4 | | 3 | | | 2 | | | 1 | | N/A | |
|  | | | **Very Confident** | | | **Confident** | | | **Somewhat Confident** | | | **Not At All Confident** | |  |
| **Please rate yourself in the following areas BEFORE the Care Transitions Program** | | | | | | | | | | | | | |  |
| I am confident that I possess knowledge needed to care for my current condition(s) | | | 4 | | | 3 | | | 2 | | | 1 | |  |
| I am confident that I possess skills needed to care for my current condition(s) | | | 4 | | | 3 | | | 2 | | | 1 | |  |
| I am confident that I could recognize changes in my current health condition(s) | | | 4 | | | 3 | | | 2 | | | 1 | |  |
| I am confident that I could take the appropriate action when I identify changes in my health condition(s) | | | 4 | | | 3 | | | 2 | | | 1 | |  |
| **Rate yourself in the following areas AFTER the Care Transitions Program** | | | | | | | | | | | | | | |
| I am confident that I possess knowledge needed to care for my current condition(s) | | | | 4 | | | 3 | | | 2 | | 1 | |  |
| I am confident that I possess skills needed to care for my current condition(s) | | | | 4 | | | 3 | | | 2 | | 1 | |  |
| I am confident that I could recognize changes in my current health condition(s) | | | | 4 | | | 3 | | | 2 | | 1 | |  |
| I am confident that I could take the appropriate action when I identify changes in my health condition(s) | | | | 4 | | | 3 | | | 2 | | 1 | |  |

| **Would you recommend the program to others?** | **🞏** | **YES** | **🞏** | **NO** |  |
| --- | --- | --- | --- | --- | --- |

**Please Explain**:

**What did you like best about the Care Transitions Program**?

____________________________________________________________________________________

**What did you like least about the Care Transitions Program**?
